# Building on Existing Technology to Enhance Remote Assessments of Brain Health

**DOI:** 10.1093/geroni/igab046.528

**Published:** 2021-12-17

**Authors:** Lydia Bazzano, Camilo Fernandez, Philip Hwang, Ileana DeAnda-Duran, Enejda Senko, Honghuang Lin, Vijaya Kolachalama, Rhoda Au

**Affiliations:** 1 Tulane University, New Orleans, Louisiana, United States; 2 Boston University, Boston, Massachusetts, United States

## Abstract

Tablets, smartphones, linked devices have features such as high-fidelity microphones, accelerometers, GPS locators, and gyroscopes can be used to capture brain health-related data. Collection of data remotely is especially important given the vulnerability of older adults to COVID and the need to protect from such exposure. As part of an American Heart Association/Gates Venture Strategically Funded Network, a number of remote assessments are being deployed to capture information related to brain health in a subset of the Bogalusa Heart Study cohort (mean age 51.4, SD 5.3). The Linus Health Platform includes applications that measure cognitive abilities, and collect digital voice features and phone sensor data that can be derived into surrogate measures of cognitive function and mood. A readily available suite of games (Lumosity) is also being used to assess cognitive health. These devices and applications offer a largely unexplored opportunity for acquiring and assessing data related to brain health.

